# Global trends in antimicrobial use in aquaculture

**DOI:** 10.1038/s41598-020-78849-3

**Published:** 2020-12-14

**Authors:** Daniel Schar, Eili Y. Klein, Ramanan Laxminarayan, Marius Gilbert, Thomas P. Van Boeckel

**Affiliations:** 1 Spatial Epidemiology Laboratory, Université Libre de Bruxelles, 1050 Brussels, Belgium; 2 Center for Disease Dynamics, Economics & Policy, Washington, DC 20005 USA; 3 Princeton Environmental Institute, Princeton University, Princeton, NJ 08544 USA; 4 Fonds National de la Recherche Scientifique, 1000 Brussels, Belgium; 5 Institute for Environmental Decisions, ETH Zurich, 8006 Zurich, Switzerland

**Keywords:** Environmental impact, Antimicrobial resistance

## Abstract

Globally aquaculture contributes 8% of animal protein intake to the human diet, and per capita consumption is increasing faster than meat and dairy consumption. Reports have documented antimicrobial use in the rapidly expanding aquaculture industry, which may contribute to the rise of antimicrobial resistance, carrying potential consequences for animal-, human-, and ecosystem-health. However, quantitative antimicrobial use across a highly diversified aquaculture industry is not well characterized. Here, we estimate global trends in antimicrobial use in aquaculture in 2017 and 2030 to help target future surveillance efforts and antimicrobial stewardship policies. We estimate antimicrobial use intensity (mg kg^−1^) for six species groups though a systematic review of point prevalence surveys, which identified 146 species-specific antimicrobial use rates. We project antimicrobial use in each country by combining mean antimicrobial use coefficients per species group with OECD/FAO Agricultural Outlook and FAO FishStat production volumes. We estimate global antimicrobial consumption in 2017 at 10,259 tons (95% uncertainty interval [UI] 3163–44,727 tons), increasing 33% to 13,600 tons in 2030 (UI 4193–59,295). The Asia–Pacific region represents the largest share (93.8%) of global consumption, with China alone contributing 57.9% of global consumption in 2017. Antimicrobial consumption intensity per species group was: catfish, 157 mg kg^−1^ (UI 9–2751); trout, 103 mg kg^−1^ (UI 5–1951); tilapia, 59 mg kg^−1^ (UI 21–169); shrimp, 46 mg kg^−1^ (UI 10–224); salmon, 27 mg kg^−1^ (UI 17–41) and a pooled species group, 208 mg kg^−1^, (UI 70–622). All antimicrobial classes identified in the review are classified as medically important. We estimate aggregate global human, terrestrial and aquatic food animal antimicrobial use in 2030 at 236,757 tons (95% UI 145,525–421,426), of which aquaculture constitutes 5.7% but carries the highest use intensity per kilogram of biomass (164.8 mg kg^−1^). This analysis calls for a substantial scale-up of surveillance capacities to monitor global trends in antimicrobial use. Current evidence, while subject to considerable uncertainties, suggests that for some species groups antimicrobial use intensity surpasses consumption levels in terrestrial animals and humans. Acknowledging the fast-growing nature of aquaculture as an important source of animal nutrition globally, our findings highlight the urgent need for enhanced antimicrobial stewardship in a high-growth industry with broad links to water and ecosystem health.

## Introduction

Aquaculture is a rapidly growing and important source of animal protein nutrition, particularly in low- and middle-income countries (LMICs). Aquatic animals represent 17% of animal protein consumed globally, and for over 40% of the world’s population, fish contribute nearly 20% of per capita animal protein consumed^1^. Reflecting dietary transitions, global food fish consumption growth (3.2%) has outpaced growth in consumption of meat from all terrestrial animal production sectors combined (2.8%), with the exception of poultry^1,2^.

Since 2001, global aquaculture has grown by 5.8% annually, driven primarily by increased demand for animal protein in fast-growing economies. Aquaculture now accounts for nearly half of the global supply of fisheries products for human consumption^1^. Asia contributes nearly 90% of global aquaculture production. In 2016, China alone accounted for 61% of global production^1^. China’s aquaculture production output represents approximately one-third of the human consumption of all capture and aquaculture fisheries products worldwide^1^.

Globally, rising demand for animal source nutrition is being met with a transition to increasingly intensive animal production systems^3,4^. This transitional period is typically characterized by an emphasis on production volume that precedes the adoption of farm biosecurity, hygiene and management standards. In this context, non-therapeutic antimicrobial use may serve to increase growth and substitute for good animal husbandry practices^5,6^. The current estimate of antimicrobial use in terrestrial food producing animals substantially exceeds human use^5^. Terrestrial food producing animal antimicrobial use is expected to grow considerably by 2030, particularly in countries with fast-growing economies like Brazil, Russia, India, China and South Africa (BRICS)^7^.

Increasing use of antimicrobials in humans and food producing animals is driving antimicrobial resistance^8^, which is amongst this era’s defining global health challenges. Rising incidence of antimicrobial resistant pathogens of animal production significance also increase treatment failure rates, undermining sustainable food animal production and animal welfare.

In aquaculture, production intensification and increasing incidence of aquatic animal pathogens are similarly driving antimicrobial use^9–11^ and antimicrobial resistance^12^ across a diversity of farmed aquatic species. Compared with antimicrobial use in terrestrial food animal production, application of antimicrobials in aquaculture provides a potentially wider environmental exposure pathway for drug distribution through water with important ecosystem health implications^9,13–15^. Antimicrobial residues in the aquatic environment alter the environmental microbiome and, consequently, ecosystem regulatory, provisioning and supporting capacities^16,17^. In addition to disease regulation, the aquatic environmental microbiome’s function in nutrient cycling, sustaining biodiversity, carbon sequestration, and freshwater availability remain important research inquiries^18^.

Further, aquaculture settings utilizing antimicrobials may serve as reservoirs for antimicrobial resistance genes, providing routes for human and animal exposure to antimicrobial resistant bacteria^11,13,17,19,20^. Although the mechanisms and directionality of transference can be challenging to establish, shared antimicrobial resistance genes encoded on mobile genetic elements in pathogens isolated from humans, terrestrial and aquatic animals, and the environment point toward pathways for movement of resistance genes across compartments^10,14,17,19,20^. Such pathways are illustrated by a putative aquaculture origin for the emergence and dissemination of select plasmid-mediated colistin resistance genes^21,22^.

Multiple geographically circumscribed studies have reported on quantities of antimicrobials used across different production systems^23–26^. These are complemented by a robust set of qualitative reviews yielding important analysis and insights on aquaculture antimicrobial use and its influencing factors^13,27–33^. However, the levels and patterns of antimicrobial use in aquaculture globally remain largely undocumented, limiting application of targeted interventions and policies promoting sound antimicrobial stewardship in a high-growth industry.

In this study we present an analysis of global antimicrobial consumption trends in aquaculture. We estimate current antimicrobial use and project use to 2030 by combining species-specific antimicrobial use coefficients from a systematic review of point prevalence surveys with aquaculture production volumes. We compare these trends with previously published data on antimicrobial use in humans and terrestrial food producing animals. The estimates presented here provide an initial foundation upon which future studies will be able to build and refine in directing iterative enhancements in antimicrobial stewardship policies.

## Methods

Baseline antimicrobial consumption and projected growth through 2030 were calculated by application of species-specific antimicrobial use coefficients to current and projected aquaculture production by species. We conducted a systematic review of peer-review and grey literature for antimicrobial use point prevalence surveys in aquaculture between 2000 and 2019, using three primary search term categories: “antimicrobial” (antimicrobial; antibiotic; veterinary medicine); “use” (use; usage; consumption; amount; quantity); and “aquaculture” (aquaculture; aquatic; fish; shellfish; marine; freshwater). We identified 25 studies representing 12 countries (Supplementary Fig. S1, Tables S1, S3) constituting 146 biomass-adjusted use rates, and from which species-specific mean antimicrobial use coefficients in milligrams per kilogram of aquatic animal biomass were obtained. Antimicrobial use coefficients by drug class were similarly obtained for analysis of use trends by class.

### Aquaculture baseline and projections

Baseline 2017 and ten-year (2018–2027) aquaculture production projections were obtained from the OECD/FAO Agricultural Outlook^34^ for 33 countries, the 28 member states of the European Union as a block, and in aggregate for 232 countries and territories comprising six regions and a global figure (Supplementary Table S2). Corresponding production statistics in 2017 by country, region, and for five species categories—catfish, shrimp, salmon, tilapia, and trout—were collected from FAO FishStat^35^. In aquaculture, antimicrobials are primarily delivered through feed for both therapeutic and non-therapeutic use^10^. As bivalve filter feeders, molluscs are unlikely to be the recipient of mass administration of antimicrobials and were excluded. For each country or region, the difference between the total production figure and the sum of the five species categories was assigned to a sixth category, “pooled.” The 2017–2027 compound annual growth rate generated projected production figures from 2028 to 2030 (Supplementary Protocol S1). Modified total production projections excluding molluscs in each country or region were derived through interpolation, by applying the proportion (total excluding molluscs/total) from 2017 to each subsequent year through 2030. A similar process was then used to obtain species-specific projections, applying the 2017 proportion of each species to the modified total in each country or region to that geography’s modified total for every year through 2030.

### Uncertainty

Mean antimicrobial use coefficients in mg kg^−1^ were calculated for each of the six species categories by calculating the mean of the log10 transformed species-specific use rates from the point prevalence surveys. The rates were log10 transformed to normalize their distributions. The 95% uncertainty intervals (UI) were calculated by applying the standard error around the mean (e.g. mean ± t*SE), where *t* is the value from the *t*-distribution for a given uncertainty interval with given degrees of freedom, and SE is the standard error. These log10 transformed mean rates and their 95% UI by species were then back transformed to generate mean species-specific antimicrobial use coefficients and their 95% UI. These coefficients were then applied to the production figures for each country or region through 2030 to generate annual antimicrobial use totals and 95% UI high and low bounds by species, and summed across all species categories to calculate annual use totals and 95% UI by country and region (Supplementary Protocol S2).

A sensitivity analysis was also performed to identify outliers. Antimicrobial use coefficients for each point prevalence survey were sequentially excluded from the calculation of the mean. The resultant mean coefficients were compared with the mean of the full list of surveys. A similar analysis was performed using mean coefficients by country. The deviation from the mean of the full complement of surveys with each survey individually removed was assessed (Supplementary Fig. S2). Point prevalence surveys, which, when removed, resulted in a deviation from the mean of the full complement of surveys (n = 146) exceeding 2% were omitted from further analyses (n = 5).

### Aggregate global consumption

Antimicrobial consumption from terrestrial food animals and baseline estimates from humans were obtained from Van Boeckel et al.^5^ and Klein et al.^36^, respectively. Consumption trends from terrestrial food animal species were adjusted to reflect revised animal biomass projections from 2017 to 2030. Human biomass projections were derived from the product of United Nations population estimates^37^ and global average human body mass^38^. The uncertainty interval for aggregate global consumption was derived from the sum of the 95% UI lower bounds for terrestrial and aquatic food animal species plus the baseline human use estimate; and sum of the 95% UI upper bounds for terrestrial and aquatic food animal species plus the continued growth human use estimate.

## Results

Global antimicrobial consumption in aquaculture in 2017 was estimated at 10,259 tons (95% uncertainty interval 3163–44,727 tons). From this baseline, global antimicrobial consumption is projected to rise 33% to 13,600 tons (95% UI 4193–59,295) by 2030. The Asia–Pacific region accounts for the overwhelming majority (93.8%) of global consumption, and this percentage is projected to remain stable throughout 2017–2030. Africa (2.3%) and Europe (1.8%) are the second and third highest consuming regions, respectively, in 2017. While Europe’s share of global consumption is projected to decrease to 1.7% by 2030, Africa’s share of global consumption is projected to rise 13% and reach 2.6% by 2030. Africa and Latin America exhibit the largest relative increase in consumption, 50.9% and 50.6%, respectively between 2017 and 2030 (Supplementary Fig. S3).

**Figure 1:**
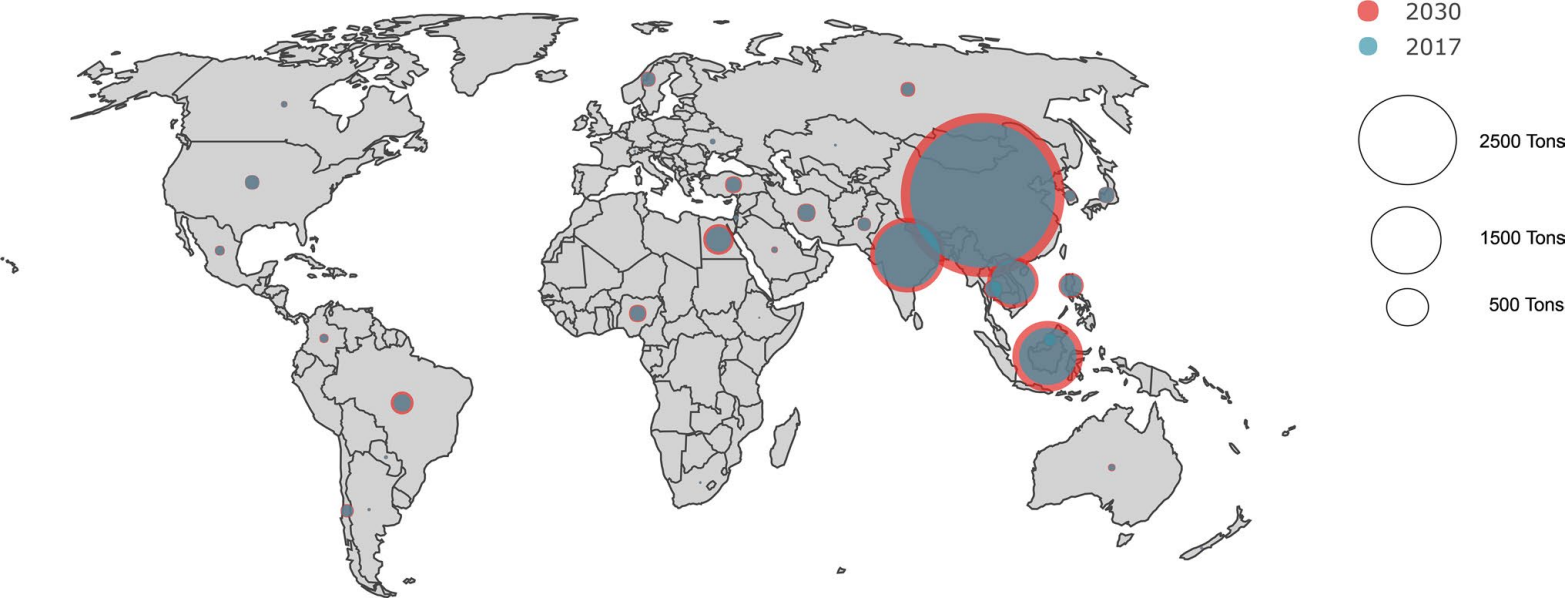
Antimicrobial consumption in aquaculture in 2017 and 2030 (projected, Supplementary material).

The four countries with the largest share of antimicrobial consumption in 2017 were all in the Asia–Pacific region: China (57.9%), India (11.3%), Indonesia (8.6%), and Vietnam (5%) (Fig. 1). These countries are projected to remain the largest consumers of antimicrobials in 2030, with China’s share decreasing marginally to 55.9%, India unchanged, and Indonesia’s and Vietnam’s share increasing to 10.1% and 5.2%, respectively. These four countries also represented the largest share of aquatic animal production output, excluding molluscs, in 2017: China (51.2%); India (9.9%); Indonesia (9.8%); and Vietnam (5.7%). The countries with the largest projected relative increase in consumption between 2017 and 2030 were Brazil (94%), Saudi Arabia (77%), Australia (61%), Russia (59%) and Indonesia (55%) (Supplementary Fig. S3).

### Antimicrobial consumption trends by species groups

**Figure 2:**
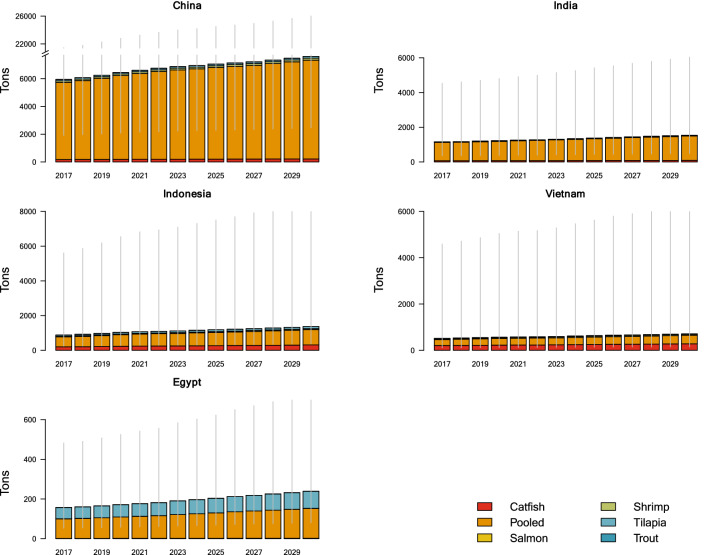
Projected antimicrobial use (tons) in aquaculture by species by 2030 in the five highest-consuming countries. The China panel y-axis is broken at 8000 and resumes at 20,000 tons. Error bars represent the 95% uncertainty intervals for the annual total use.

Five species groups were established in our study: catfish, shrimp, salmon, tilapia, and trout. A sixth group, “pooled”, with a mean use coefficient comprised of 12 species from 11 surveys, was created to capture antimicrobial consumption across a diversity of other species, or from studies where the species could not be individually identified (Supplementary material). Molluscs were excluded from this study. In 2017, the largest share of antimicrobial consumption was associated with the multi-species “pooled” category (84.1%). Among the individual species groups, 8.3% of global antimicrobial consumption was attributable to catfish, 3.4% to tilapia, 2.7% to shrimp, 0.8% to trout, and 0.7% to salmon. The relative proportion of each species group was stable through 2030 (Fig. 2). Mean antimicrobial use coefficients across the six species groupings were: in the “pooled” category, 208 mg kg^−1^ (95% UI 70–622); catfish, 157 mg kg^−1^ (UI 9–2751); trout, 103 mg kg^−1^ (UI 5–1951); tilapia 59 mg kg^−1^ (UI 21–169); shrimp, 46 mg kg^−1^ (UI 10–224); and salmon, 27 mg kg^−1^ (UI 17–41).

The influence of production system on antimicrobial use was assessed using a one-way analysis of variance (ANOVA) test, which revealed no significant association (P = 0.543) between production system intensity and use when comparing intensive, semi-intensive, and mixed systems (Supplementary Fig. S8). Notably, production under extensive rearing conditions was identified in only two of 146 surveys, both of which were omitted following the sensitivity analysis.

### Trends by antimicrobial class

Globally, the most commonly used classes of antimicrobials were, by frequency of use, quinolones (27%), tetracyclines (20%), amphenicols (18%), and sulfonamides (14%). Antimicrobials listed by the World Health Organization (WHO) as critically important and highly important for human medicine^39^ accounted for 35% and 61% of use, respectively, across surveys (Supplementary Fig. S4). Mean use coefficients were highest for first generation cephalosporins at 85 mg kg^−1^, lincosamides at 33 mg kg^−1^, and tetracyclines at 23 mg kg^−1^ (95% UI 11–45), although uncertainty intervals could not be generated for the former two classes due to limited sample sizes.

### Antimicrobial consumption from humans, terrestrial animals, and aquaculture

**Figure 3:**
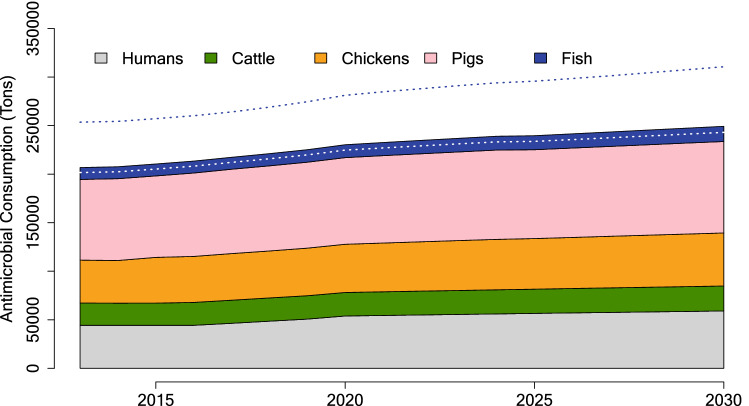
Global antimicrobial consumption, 2013–2030. Dotted lines represent the 95% uncertainty interval for fish.

By 2030, global antimicrobial use from human, terrestrial and aquatic food producing animal sectors is projected to reach 236,757 tons annually (95% UI 145,525–421,426) (Fig. 3). Proportion of use across sectors remains relatively consistent through 2030, when human use (48,608 tons), terrestrial food producing animal use (174,549 tons), and aquatic food producing animal use (13,600 tons) represent 20.5, 73.7, and 5.7% of global consumption, respectively.

## Discussion

### Global trends

At current rates, global antimicrobial consumption in aquaculture is expected to increase 33% between 2017 and 2030. These estimates are associated with considerable uncertainty and relatively wide uncertainty intervals due to the scarcity of point prevalence surveys on antimicrobial use in aquaculture. Global trends in antimicrobial use in aquaculture are heavily influenced by the expansion of aquaculture in Asia, and particularly in China (Fig. 2). Since 1991, China alone has accounted for more annual farmed fish output by weight than all other countries combined^1^.

Collectively, the five countries with the largest projected relative increase in antimicrobial consumption between 2017 and 2030 (Supplementary Fig. S3) account for only 11.5% of global antimicrobial consumption in 2030, indicating that, with the exception of Indonesia, the countries with the fastest antimicrobial consumption growth will remain the minority contributors to global consumption projected by 2030.

Several localized antimicrobial use point prevalence surveys^23–26,40,41^ (Supplementary Table S3) and reviews^28,30,33,42^ have been conducted, providing important individually detailed use profiles by species and location. Our study provides current global antimicrobial use estimates and projections through 2030, complementing these studies and placing aquaculture use in the broader context of use in human and terrestrial food animal production sectors.

At a country-level, few estimates of antimicrobial use in aquaculture have been produced. Although this limits comparisons of our estimates with country-level use, our antimicrobial use estimates for aquaculture are within the range of a 2013 domestic consumption estimate from China^43^, and approximately 40% of the low bound from a 2002 estimate in the United States^44^.

On an equivalent biomass basis, estimated antimicrobial consumption in 2017 from aquaculture (164.8 mg kg^−1^) is 79% higher than human consumption (92.2 mg kg^−1^) and 18% higher than terrestrial food producing animal consumption (140 mg kg^−1^), shifting to 80% higher than human (91.7 mg kg^−1^) consumption and remaining 18% higher than terrestrial food producing animal consumption projected in 2030.

### Drug classes and species

The most commonly used antimicrobial classes observed in this study (Fig. S5)—quinolones, tetracyclines, amphenicols, and sulfonamides—were consistent with a previous review of antimicrobials used in the 15 highest aquaculture-producing countries^13^. Countries with export-oriented production largely follow the regulatory structures and maximum residue limits (MRLs) established by the European Union and United States. The four countries with the highest antimicrobial consumption identified in this study (China, India, Indonesia, and Vietnam), for example, have adopted European Union MRLs to meet export requirements^13^. Notably, enforcement of regulations remains a challenge in many countries, and parallel production systems serving domestic and export markets may encourage differing use practices^27^.

All of the classes of antimicrobials identified in our systematic review of point prevalence surveys are classified by the World Health Organization as important for human medicine. Classes assigned to the top two classification tiers—highly important and critically important antimicrobials for human medicine—collectively represented 96% of all use (Supplementary Fig. S4). This finding is of particular concern given that few alternatives to these drug classes exist^45^. It further raises the prospect of antimicrobial use in aquaculture driving resistance trends in aquatic environments, with implications for transfer of resistance genes across bacterial species^10,13,27^. Such transfers are ecological in nature and are thus challenging to document^46^. However, the transmission of resistance genes across bacteria capable of spanning the aquatic–environment–human interface with corollary public health impact, as has been described in terrestrial food producing animal settings^47,48^, has been suggested^9–11,13,19–22,27,49,50^. This dynamic may be particularly important in areas reliant upon untreated water sources and with higher rates of consumption of raw fisheries products.

Salmon accounted for the lowest antimicrobial use across the six species groups, although use coefficients in commercial salmon production varied by several orders of magnitude across countries^26,40,51,52^. Comparatively low antimicrobial consumption rates in salmon in some countries could be reflective of an intensified and highly structured commercial salmon production industry operating with the benefit of vaccines for prevention of production significant pathogens. In intensified and vertically integrated terrestrial animal production, such commercial interests may operate under improved husbandry and management conditions, including high vaccine coverage rates and use of specific pathogen free broodstock, with reduced reliance on prophylactic antimicrobial use, which may parallel conditions in the salmon industry in select countries.

Several countries have experienced dramatic reductions in antimicrobial use rates following introduction of vaccination and improved management and husbandry programs^10,27,29,53^, serving as important antimicrobial stewardship models. Future strategies aimed at strengthening aquaculture production without pharmaceutical interventions may leverage advancements in bacteriophage therapy, pre- and probiotics, and CRISPR-Cas genome editing^10^. Identifying solutions that can be implemented and economically self-sustaining in low- and middle-income country settings currently representing the substantial majority of aquaculture production is imperative.

## Limitations

In the absence of comprehensive, standardized antimicrobial use data, our study relies upon a relatively limited collection of antimicrobial use point prevalence surveys. Despite a Chinese language search of the China National Knowledge Infrastructure (CNKI) database, surveys on antimicrobial use in China are under-represented in our study, constituting an important knowledge gap on antimicrobial use in the world’s largest aquaculture producing nation. This limitation is particularly acute for some of the most commonly farmed fish species, such as the freshwater Cyprinidae family of species that includes carp^27^.

Our antimicrobial consumption projections are subject to wide uncertainty intervals that likely reflect both the limited availability of surveys, from which projections were generated, and the diversity of global aquaculture production systems, practices and species. The diversity of farmed aquatic animal species greatly exceeds terrestrial food animal producing species. In 2016, 558 distinct aquatic animal species items were commercially farmed worldwide^1^. Although a smaller subset of 27 species groups accounted for 90% of global aquaculture production in 2016, by comparison, only three species groups—chickens, pigs and cattle—contributed to an equivalent level of terrestrial food animal production^1,54^. Such a diversity of farmed aquatic species—compounded by polyculture production systems—presents substantial variability and challenges documentation of antimicrobial use. Currently, antimicrobial use is poorly documented even for those species of greatest production significance. Carp represent more than one-third of global aquaculture production tonnage by species^1^, however, only one survey of farms in Vietnam^55^ explicitly identified carp amongst the species for which antimicrobial use data were collected. Antimicrobial use coefficients by species, particularly for the “pooled” category that comprises data representing 12 aquatic animal species, may therefore not approximate actual use rates in select species. The use coefficient for this “pooled” category should rather be interpreted as approximating a mean use intensity across a broad spectrum of farmed aquatic animals.

There are additional sources of uncertainty in our projections. Endemic and emerging aquatic animal pathogens^56^ can be expected to impact both aquaculture output and antimicrobial use rates, with implications for antimicrobial consumption modeling. Further, dual structured production systems employing differing antimicrobial use practices for export and domestic markets are not uncommon in LMICs^27^. The data on the relative prevalence of these systems is not sufficiently granular to incorporate into our projections.

Finally, without data capturing temporal trends in species-specific antimicrobial use, we assumed that the mean use coefficients by species remain constant between 2017 and 2030. As a consequence, variability in antimicrobial consumption solely reflects the growth in aquaculture production in each country or region through 2030. Despite these limitations, our estimates provide a starting point to help frame a discussion outlining near-term priorities to enhance antimicrobial use data collection.

The antimicrobial use estimates in this study may be conservative for two reasons. First, 96.5% of surveys were conducted in World Bank-classified high- and upper middle-income countries^57^, where antimicrobial resistance has been a concern for decades, and active antimicrobial stewardship campaigns and regulatory structures are present. Second, the point prevalence surveys from which our antimicrobial use coefficients originate generally document individual therapeutic or metaphylactic application events on farm at a single point in the production cycle, rather than capturing multiple applications and total use across the cycle. Further, in some regions, swine and poultry manure effluents are commonly applied to aquaculture waters under integrated farming conditions^58^. Our analysis likely underestimates such indirect antimicrobial application to cultured species and their aquatic environment, with implications for selection pressures driving resistance.

A confluence of trends in animal-source nutrition availability could push accelerated rates of aquaculture growth in the near-term. Increasing ocean acidification and warming has been projected^59,60^ to represent a net negative impact on capture fisheries output, which have plateaued over the last two decades^1^. And terrestrial animal epizootics, such as African swine fever in Asia, are constraining terrestrial animal-source nutrition supply^61^. In this context, an acute re-orientation of protein demand to aquatic animal-source food products could be expected to drive increased aquaculture production output. Under business as usual conditions, this would lead to an increase in antimicrobial consumption in aquaculture. Such trends could be significant in areas with widespread availability of—and unrestricted access to—antimicrobials^23^.

Our study underscores the urgent need for standardized antimicrobial use surveillance in the aquaculture industry. Experience from countries implementing antimicrobial stewardship initiatives cite the establishment of antimicrobial use surveillance structures as the foundation for identifying risk and targeting interventions^62–64^. Robust surveillance data (1) facilitates identification of sectors and production contexts where either inappropriate use or lack of access would benefit from rebalancing; (2) enables the establishment of time-bound, measurable consumption targets and monitoring progress toward achieving these targets; and (3) when paired with resistance data, generates additional insight into the association between patterns of consumption and antimicrobial resistance trends. A tiered approach to surveillance of antimicrobial consumption permits utilization of existing sales channel data to direct enhanced stewardship policies while structures are developed to produce iteratively more granular, farm-level consumption data^65^. As a function of potentially higher rates of off-label use of antimicrobials in aquaculture—particularly in developing country contexts—sales data, however, may currently under-represent consumption. Labelled indications for therapeutic use in primary aquaculture species will improve attribution to—and characterization of—aquaculture use.

The study uses current evidence to draw a first assessment of the global trends in antimicrobial use in aquaculture. Aquaculture-associated antimicrobial consumption remains a minority share of total global consumption through 2030. However, high industry growth rates, shifting dietary preferences and transitions to intensified production without corresponding management changes may drive increasing antimicrobial use in aquaculture relative to other sectors. Increases in use may be particularly significant in geographies with nascent antimicrobial consumption surveillance, regulatory and enforcement capacities. Our findings call for urgent strengthening of surveillance for antimicrobial consumption and enhanced understanding of antimicrobial resistance transmission risk across the aquatic animal–environment–human interface, with application of targeted policies and regulatory structures promoting antimicrobial stewardship and antimicrobial efficacy as a shared global resource.

## Supplementary Information

Supplementary Information.
