# Supplementary material for: Global trends in antimicrobial use in aquaculture
Source: Sci Rep. 2020 Dec 14;10:21878. doi: 10.1038/s41598-020-78849-3 (PMC7736322; doi:10.1038/s41598-020-78849-3)
Supplement: Supplementary file 1 — Supplementary Information. [file 41598_2020_78849_MOESM1_ESM.docx]

**Supplementary Material**

Global Trends in Antimicrobial Use in Aquaculture

Daniel Schar, VMD^a,*^, Eili Y. Klein, PhD^b^, Ramanan Laxminarayan, PhD ^b,c^, Marius Gilbert, PhD ^a,d^, Thomas P. Van Boeckel, PhD ^b,e^

^*^To Whom correspondence may be addressed: Daniel Schar

Email: dlschar@gmail.com

**This PDF file includes:**

Supplementary text

Figures S1 to S8

Tables S1 to S3

References for supplementary text reference citations

**Supplementary Material Text**

*Systematic Review of Antimicrobial Use Point Prevalence Surveys*

**Search Methods.** Point prevalence surveys were identified through peer-reviewed and grey literature database searches conducted between December 2018 and November 2019 (table S1). Search terms included a series of three primary categories: “antimicrobial” (antimicrobial; antibiotic; veterinary medicine); “use” (use; usage; consumption; amount; quantity); and “aquaculture” (aquaculture; aquatic; fish; shellfish; marine; freshwater). Search categories, and search terms within categories, were joined using the Boolean operators AND and OR respectively. Specific phrases (“veterinary medicine”) were enclosed in double quotes, and search terms were prefix or suffix-truncated with the wildcard asterisk, as relevant, to expand the breadth of search terms.

Recognizing the substantial share of global aquaculture production originating in China,^1^ a Chinese-language search in the China National Knowledge Infrastructure (CNKI) database was conducted in March 2019 (table S1). Search terms mirrored those in the primary English-language search, specifically “antimicrobial” (‘抗生素’ ‘抗菌素’ ‘兽药’ ‘兽用药’ ‘兽用抗生素’); “use” (’使用’ ‘用量’ ‘消费’); and “aquaculture” (’水产养殖’ ‘水生’ ‘鱼’ ‘贝壳’ ‘贝类’ ‘海洋’ ‘淡水’).

Search categories and terms were tailored to individual databases to achieve optimal search sensitivity and specificity. Duplicate records were excluded using a reference management platform (Mendeley). Screening was undertaken in three stages, with an initial review of titles, followed by abstracts, and finally full text records. The PRISMA Statement^2^ on conducting systematic reviews and meta-analyses guided each stage of the review (figure S1).

**Eligibility and Exclusion Criteria.** Records met eligibility criteria for inclusion in the systematic review if they contained quantitative antimicrobial use data from aquaculture settings (farm surveys, sales, prescription, or distribution) published between 2000 and 2019. Records meeting eligibility criteria for antimicrobial use were excluded if no methodology or source for derivation of figures was provided; or source was another publication included in the review; or data was extrapolated from production volumes, estimated disease frequency, and standard dosing indications; or data was collected exclusively from a research protocol. One record consisted of a case report of therapeutic antimicrobial application in individual Australian lungfish from a zoological collection and was excluded from the quantitative synthesis as the animals were not intended to enter the human food chain.

**Data Extraction and Synthesis.** The data extracted from the point prevalence surveys included: a digital object identifier; author name; publication date; country; geographic coordinates; start and end dates for sampling campaign; species; water type; data source; culture system; production stage; drug class; active pharmaceutical ingredient (API); API quantity (mg); API total use (mg); biomass (kg); Mg·kg^−1^ ; and remarks/notes and author email variables. Four additional variables were added for synthesis or annotation of extracted data: geographic coordinates comments; administrative level; species aggregation; and culture system comments.

All fish species were grouped into six categories—catfish, salmon, shrimp, tilapia, trout, and the pooled category. Surveys were assigned to the pooled category if no species was specified; or if the species referenced could not be included in the other five categories; or multiple species were identified. For surveys specifying combinations of antimicrobials (e.g. sulfadiazine-trimethoprim or lincomycin/streptomycin), the class was assigned based on the predominant API by weight in the product. Where biomass data was presented in the record, that data was included into our database, as denominator; alternatively, where the study reports national-level quantitative API, but does not report a population at risk for treatment, biomass for the relevant production system and time period from FAO FishStat^3^ was used to calculate the mg·kg^−1^. Start and end dates with months identified were set at the 15th of the month; annual dates were set from January 1^st^ of the commencing year to December 31^st^ of the concluding year. The assignment of culture system follows the definition for "extensive," "semi-intensive," and "intensive" in Edwards.^4^ The full protocols for data extraction and synthesis are presented in the database legend (doi: 10.5281/zenodo.4305096).

**Table S1: Characteristics of systematic review**

| **Database** | **Records Identified** | **Records after auto and manual de-duplicating** | **Abstracts screened for eligibility and exclusion criteria** | **Full text articles reviewed** | **Articles included for data extraction and analysis** |
| --- | --- | --- | --- | --- | --- |
| PubMed (n=2685), Web of Science (n=494), Scopus (n=1194) | 4373 | 3847 | 251 | 127 | 24 |
| World Health Organization Library Database (WHO LIS) | 19 | 19 | 3 | 1 | 0 |
| Food and Agriculture Organization of the United Nations (AGRIS) | 360 | 360 | 14 | 8 | 1 |
| CGIAR FISH | 159 | 158 | 7 | 3 | 0 |
| International Food Policy Research Institute (IFPRI) | 9 | 9 | 5 | 0 | 0 |
| WorldFish | 8 | 7 | 5 | 0 | 0 |
| **Sub-Total** | 4928 | 4400 | 285 | 139 | 25 |
| China National Knowledge Infrastructure (CNKI) | 1184 | 1184 | 49 | 49 | 0 |
| **Total** | 6112 | 5584 | 334 | 188 | 25 |

*Antimicrobial sales*

**Search Methods.** As an additional source of data, nationally reported antimicrobial sales were collected through a search of country repositories housed within ministries of agriculture, food and drug administrations, or veterinary bureaus. Search categories and terms followed those described in the systematic review of antimicrobial use point prevalence surveys. An initial search was conducted using each of the 23 country websites available in chapter nine of the OIE annual report on antimicrobial agents intended for use in animals,^5^ screening available repositories for antimicrobial use or consumption data from aquaculture settings. Additional searches were conducted on a global antimicrobial consumption monitoring platform^6^ and for nine countries representing over 85% of global aquaculture production.

**Data Extraction and Synthesis.** Nationally reported sales data were manually extracted into a database (Google Sheets). The created sales database included country; antimicrobial class; API; freshwater sales (kg); marine sales (kg); total sales (kg); total sales (mg); replicates of both domestically-reported and FishStat-reported freshwater biomass (kg), marine biomass (kg), and total biomass (kg); year; mg·kg^−1^ calculated using domestically-reported biomass; mg·kg^−1^ calculated using FishStat-reported biomass; a URL link to the data; and notes.

Antimicrobial classes were extracted directly from sales reports, and assigned to “Other” where so assigned in the national report (Denmark); or where the sales were reported as combination products (Norway); or in a single instance (Bicozamycin benzoate, a 2,5-diketopiperazine member of the cyclic polypeptides) of a rarely used class in Japan. For countries reporting only total annual sales, not disaggregated by class, the class for that country in that year was assigned as “all class.” API sales for use in ornamental fish were excluded, on the assumption that such fish do not enter the food chain. In calculating the mg·kg^−1^ using FishStat-reported biomass, alignment was sought between the API sales reports and FishStat-reported biomass. For example, where API sales were reported for “fish” or “finfish” or “farmed fish,” FishStat-reported biomass excluding crustaceans and molluscs was used.

Sales data from eight countries were obtained but were not reported by species; six countries reported sales by class (figure S6). The results of this search are largely consistent with the number of countries (n = 9) identified in the OIE report as providing quantitative aquaculture-disaggregated sales data, however, the OIE does not make member state-reported data public and does not identify these countries by name.

Country and time-paired analyses of use coefficients from the point prevalence surveys and the national sales data revealed moderate dispersion but also a positive association when grouped across all classes (figure S7). However, limited availability of national sales data and reporting of this data in aggregate for all species precluded their utility in modeling projected use.

Data analysis was conducted in R (version 3.5.3). Point prevalence surveys reporting zero antimicrobial use were log transformed log10(x+1). Antimicrobial use estimates and projections to 2030 were generated using the following protocols.

**Protocol S1. Aquaculture Production Biomass by Species.** The OECD FAO Agricultural Outlook^7^ forecasts were used to obtain projected aquaculture production figures by country and region from 2017 through 2027. The compound annual growth rate between 2017 and 2027 was used to obtain projected production figures through 2030. The Outlook provides these figures for 33 countries plus the 28 member states of the European Union as a block, and in aggregate for countries and territories comprising each of six regions (table S2). The FAO FishStat database^3^ was used to obtain aquaculture production figures by species for 2017 in each of the 33 countries and the 28 member states of the European Union as a block, for which future projections were available from the OECD FAO Outlook, as well as by region for Africa, Asia, Europe, Latin America, North America, and Oceania. For FAO FishStat regional figures, production from countries in each region were matched with those identified as comprising the aggregated regional figures in the OECD FAO Outlook. This match was completed for all countries in Africa, North America, and Oceana, and for 64/73 countries in Asia, 45/46 countries in Europe, and 46/47 countries in Latin America. Countries/territories for which no FAO FishStat data were available are noted in table S2.

Production figures from 2017 in metric tons for each of five species categories—catfish, shrimp, salmon, tilapia, and trout—and a total production figure were obtained from FAO FishStat. The total production figure excluded molluscs, both because no point prevalence surveys were identified in the systematic review that included molluscs; and, as medicated feed is the primary delivery mechanism for antimicrobial use in aquaculture, bivalve molluscs as filter feeders are relatively less likely to receive mass administration of antimicrobials. For each country or region, the difference between the total production figure and the sum of the five species categories was assigned to a sixth category, “pooled.”

**Protocol S2. 2017 Baseline Antimicrobial Use and Projected Use in 2030.** Antimicrobial use projections through 2027 were derived as follows. First, using 2017 data from Protocol S1, the proportion of total production from FishStat to the OECD FAO production figure was calculated for each country or region, and subsequently applied to the OECD FAO forecasts for each year through 2030 to obtain a revised production forecast.

$${P_{rev}}_{j,y}= {{(FS}_{2017}/OF}_{2017})\cdot{OF}_{y}$$

Where ${P_{rev}}_{j,y}$ is the revised production forecast in country or region (*j)* and year (*y)*;${FS}_{2017}$ and ${OF}_{2017}$ are the FishStat and OECD FAO production figures, respectively, in 2017; and ${OF}_{y}$ is the OECD FAO forecast for each year (*y*) through 2030.

Second, using the 2017 FishStat data by species from protocol S1, the proportion of each of the six species categories to the total revised production forecast in each country or region was calculated, and applied to the revised forecasts for the subsequent year from step one for that country or region in each year through 2030 to obtain annual species disaggregated production figures.

$$P_{k,j,y}= \frac{{FS}_{k,y-1}}{P_{rev y-1}} \cdot P_{rev j,y}$$

Where $P_{k,j,y}$ are the production figures for species (*k)* in country or region (*j*) and year (*y*); ${FS}_{k,y-1}$ is the FishStat production figure for species (*k*) in the immediately preceding year; and $P_{rev y-1}$is the total revised production forecast in the immediately preceding year.

Third, production figures were converted from metric tons to kilograms for alignment with mean mg·kg^−1^ coefficients and their 95% uncertainty intervals (UI) calculated by applying the standard error around the mean by species for each of the six species categories derived from the point prevalence surveys. The species-specific coefficients and their 95% UI were then applied to the production figures by species from step two for each country or region in each year through 2030 to obtain antimicrobial use volumes by species.

$$R_{k,j,y}=P_{k,j,y}\cdot1000\cdot\alpha_{k}$$

Where $R_{k,j,y}$ are the antimicrobial use volumes for species (*k*) in country or region (*j)* and year (*y)*; and ($\alpha_{k}$) are the antimicrobial use coefficients for species (*k*).

Finally, the sum of the total weights of antimicrobial use across species in each country or region in each year through 2030 was calculated to derive a total annual antimicrobial use estimate with 95% UI. Total annual use in a county was defined as:

$$Consumption= \sum_{k}^{6} R_{k,j,y}$$

**Table S2. Countries represented in the OECD FAO Agricultural Outlook regional production figures**

| **Africa†** | **Asia** | **Europe** | **Latin America** | **North America** | **Oceania** |
| --- | --- | --- | --- | --- | --- |
| Algeria, Angola, Benin, Botswana, British Indian Ocean Ter., Burkina Faso, Burundi, Cabo Verde, Cameroon, Central African Republic, Chad, Comoros, Congo, Côte d'Ivoire, Dem. Rep. of the Congo, Djibouti, Egypt, Equatorial Guinea, Eritrea, Eswatini, Ethiopia, Ethiopia PDR, French Southern Terr., Gabon, Gambia, Ghana, Guinea, Guinea-Bissau, Kenya, Lesotho, Liberia, Libya, Madagascar, Malawi, Mali, Mauritania, Mauritius, Mayotte, Morocco, Mozambique, Namibia, Niger, Nigeria, Rwanda, Réunion, Saint Helena, Sao Tome and Principe, Senegal, Seychelles, Sierra Leone, Somalia, South Africa, South Sudan, Sudan, Sudan (former), Togo, Tunisia, Uganda, United Rep. of Tanzania, Western Sahara, Zambia, Zanzibar, Zimbabwe | Afghanistan, American Samoa, Bahrain, Bangladesh, Bhutan, Brunei Darussalam, Cambodia, China, Chinese Taipei, Christmas Island*, Cocos (Keeling) Islands*, Cook Islands, Democratic People's Republic of Korea, Fiji Islands, French Polynesia, GAZA*, Gaza Strip (Palestine)*, Guam, Hong Kong (China), India, Indonesia, Iran, Iraq, Japan, Johnston Islands*, Jordan, Kiribati, Korea, Kuwait, Lao People's Democratic Republic, Lebanon, Macau, Malaysia, Maldives, Marshall Islands, Micronesia (Federated States of), Mongolia, Myanmar, Nauru, Nepal, Neutral Zone*, New Caledonia, Niue, Norfolk Island, Northern Mariana Islands, Oman, Pacific Islands*, Pakistan, Philippines, Palau, Palestine Occupied Tr., Papua New Guinea, Pitcairn Islands, Qatar, Samoa, Saudi Arabia, Singapore, Solomon Islands, Sri Lanka, Syrian Arab Republic, Thailand, Timor-Leste, Tokelau, Tonga, Turkey, Tuvalu, United Arab Emirates, US Minor Outlying Islands*, Vanuatu, Viet Nam, Wallis and Futuna Islands, West Bank*, Yemen | Albania, Andorra, Belarus, Bosnia and Herzegovina, European Union -28, Faeroe Islands, Gibraltar, Holy See*, Iceland, Monaco, Montenegro, Norway, Republic of Moldova, Russian Federation, San Marino, Serbia, The former Yugoslav Republic of Macedonia, Ukraine, Switzerland | Anguilla, Antigua and Barbuda, Netherlands Antilles, Argentina, Aruba, Bahamas, Barbados, Belize, Bolivia, Brazil, Chile, Colombia, Costa Rica, Cuba, Dominica, El Salvador, Ecuador, Grenada, Guadeloupe, Guatemala, Guyana, French Guiana, Haiti, Honduras, Cayman Islands, Falkland Islands (Islas Malvinas), Turks & Caicos Islands, United States Virgin Islands, British Virgin Islands, Jamaica, South Georgia and the South Sandwich Islands*, Martinique, Mexico, Montserrat, Nicaragua, Panama, Paraguay, Peru, Puerto Rico, Dominican Republic, Saint Lucia, St. Kitts and Nevis, Saint Vincent and the Grenadines, Suriname, Trinidad and Tobago, Uruguay, Venezuela | Canada, United States | Australia, New Zealand |

†For the Africa region, the OECD FAO Agricultural Outlook database names only “North Africa, Sub-Saharan Africa.” As the Outlook data for Africa originates from FAO, the FAO FishStat countries are assumed to represent those in this database in aggregate for the Africa region and are listed here.

*Countries/territories for which no corresponding data is available in FAO FishStat

**Table S3. Data sources included in the meta-analysis**

| Author | Publication Date | Country | Aquatic Species | Reference |
| --- | --- | --- | --- | --- |
| Rico A | 2014 | Thailand | Tilapia | 10.1016/j.envpol.2014.04.002 |
| Rico A | 2013 | Bangladesh, China, Thailand, Vietnam | Shrimp, Prawn, Tilapia, Catfish | 10.1016/j.aquaculture.2013.07.028 |
| Burridge L | 2010 | Canada, Norway, Great Britain | Salmon | 10.1016/j.aquaculture.2010.05.020 |
| Fraser E | 2004 | Canada | Salmon | Can Vet J 45:309–311 |
| Pham DK | 2015 | Vietnam | Tilapia, Carp, Snakehead, Catfish, Prawn | 10.1007/s10393-014-1006-z |
| Miranda CD | 2018 | Chile | Salmon | 10.3389/fmicb.2018.01284 |
| Bravo S | 2012 | Chile | Salmon | FAO Fisheries and Aquaculture Technical Paper No. 547. Rome, FAO. http://www.fao.org/3/a-ba0056e.pdf#page=29 |
| Animal Health Products Association | 2017 | Thailand | Fish, Shrimp | http://www.vetcouncil.or.th/index.php?option=com_docman&task=doc_download&gid=962&Itemid= |
| Hedberg N | 2018 | Vietnam | Cobia, Grouper, Snapper, Spiny Lobster | 10.1016/j.aquaculture.2018.06.005 |
| Higuera-Llanten S | 2018 | Chile | Salmon | 10.1371/journal.pone.0203641 |
| Andrieu M | 2015 | Vietnam | Catfish | 10.1016/j.chemosphere.2014.06.062 |
| Schmidt AS | 2000 | Denmark | Trout | 10.1128/aem.66.11.4908-4915.2000 |
| Lillehaug A | 2018 | Norway | Salmon | 10.3354/dao03219 |
| Chen B | 2018 | China | Bullfrog | 10.1016/j.watres.2018.02.003 |
| Grave K | 2008 | Norway | Salmon, Trout, Cod | 10.1016/j.prevetmed.2007.07.002 |
| Radke B | 2018 | Canada | Salmon | PMID: 29599557 |
| Bosma RH | 2009 | Vietnam | Catfish | http://library.wur.nl/WebQuery/wurpubs/fulltext/8332 |
| Nonaka L | 2007 | Japan | Red Seabream, Convict Grouper, Red Spotted Grouper | 10.1264/jsme2.22.355 |
| Morrison DB | 2013 | Canada | Salmon | PMID: 24293677 |
| Coyne R | 2001 | Ireland | Salmon | Marine Environment and Health Series No. 3, Marine Institute https://oar.marine.ie/handle/10793/221 |
| Furushita M | 2007 | Japan | Not Stated^†^ | Journal of National Fisheries University 56(1): 85-89 http://www.fish-u.ac.jp/kenkyu/sangakukou/kenkyuhoukoku/56/01_9.pdf |
| Rodgers CJ | 2009 | Canada, Great Britain | Salmon, Trout | http://ressources.ciheam.org/om/pdf/a86/00801061.pdf |
| Cermaq | 2017 | Canada, Chile, Norway | Salmon | https://www.cermaq.com/wps/wcm/connect/cermaq/cermaq/our-company/annual-report/sustainability-report-17/cermaq-indicators-17-s |
| Mowi | 2019 | Norway, Great Britain, Ireland, Faroe Islands, Canada, Chile | Salmon | https://issuu.com/hg-9/docs/mowi_annual_report_2018_4e0dacb83168e4?e=19530043/68703955 |
| Norwegian Veterinary Institute | 2016 | Norway | Salmon, Trout, Halibut, Turbot, Cod, Char, Wolffish | https://www.vetinst.no/rapporter-og-publikasjoner/rapporter/2016/use-of-antibiotics-in-norwegian-aquaculture |

†Aggregate Japan use totals for 2001.

**Included**

**Eligibility**

**Screening**

**Identification**

Records identified through database searching (n=4373)

PubMed (2685); Web of Science (494); Scopus (1194)

Additional records identified through other sources (n=555)

WHO LIS (19); AGRIS (360); CGIAR FISH (159); IFPRI (9); WorldFish (8)

Records after duplicates removed (n=4400)

Titles screened (n=4400)

Titles excluded (n=4115)

Full-text articles assessed for eligibility (n=139)

Studies included in qualitative synthesis (n=26)

Full-text articles excluded (n=113)

- No quantitative use data (94)
- No source/methodology for data provided (9)
- Outside date range (1)
- Data collected exclusively from a research protocol (6)
- Extrapolated data (3)

Studies included in quantitative synthesis (meta-analysis) (n=25)

Abstracts screened (n=285)

Abstracts excluded (n=146)

- No quantitative use data (120)
- Not in English (2)
- Data collected exclusively from a research protocol (18)
- Not available for review (6)

Full-text excluded from quantitative synthesis (n=1)

**Figure S1.** Flow chart for systematic review^2^ and meta-analysis of quantitative antimicrobial use in aquaculture.

**Figure S2.** Sensitivity analysis to individual point prevalence surveys. The value is expressed as the percent deviation from the mean of the full set of surveys (n = 146) with each survey individually removed.

**Figure S3.** Growth in antimicrobial use between 2017 and 2030 by (A) country and (B) region.

**Figure S4.** Antimicrobial use (top panel, mg·kg^−1^ [Log 10]; and bottom panel, mg·kg^−1^) by class. Figures above the whiskers denote frequency of use (total use events [n = 236], and proportion of total use events across all point prevalence surveys). AMI, aminoglycosides; MAC, macrolides; POL, polymyxins; QUI, quinolones; AMP, amphenicols; CEP, 1^st^ and 2^nd^ generation cephalosporins; LIN, lincosamides; PEN, penicillins; SUL, sulfonamides; TET, tetracyclines; OTH, other medically important antimicrobials

**Figure S5.** Antimicrobial use by active pharmaceutical ingredient (n = 236)

**Figure S6.** Annual sales in six countries by antimicrobial class, expressed in mg·kg^−1^. CHL, Chile; JPN, Japan; FRA, France; DEN, Denmark; GBR, Great Britain; NOR, Norway.

**Figure S7.** Association between mg·kg^−1^ coefficients from point prevalence surveys and national sales. Association and linear regression for all country and time-paired use coefficients between 2000 and 2017. CHL, Chile; DEN, Denmark; GBR, Great Britain; JPN, Japan; NOR, Norway.

**Figure S8.** Antimicrobial use (mg·kg^−1^) by aquaculture production system. Assignment of production system follows the definitions for "semi-intensive," and "intensive" in Edwards.^4^

**References**

1. FAO. 2018. The State of World Fisheries and Aquaculture 2018 - Meeting the sustainable development goals. Rome. http://www.fao.org/3/i9540en/I9540EN.pdf (accessed July 30, 2019).

2. Moher D, Liberati A, Tetzlaff J, Altman DG, The PRISMA Group. Preferred Reporting Items for Systematic Reviews and Meta-Analyses: The PRISMA Statement. *PLoS Med* 2009; **6:** e1000097. doi:10.1371/journal.pmed1000097

3. FAO Fisheries and Aquaculture Department, Statistics and Information Service FishStatJ: Universal software for fishery statistical time series. Copyright 2016. Rome. http://www.fao.org/fishery/statistics/global-aquaculture-production/query/en (accessed July 30, 2019).

4. Edwards, P. 1993. Environmental issues in integrated agriculture-aquaculture and wastewater-fed fish culture systems. p. 139-170. In R.S.V. Pullin, H. Rosenthal, and J.L. Maclean (eds.) Environment and Aquaculture in Developing Countries. ICLARM Conf Proc 31. 359 p. http://www.fao.org/3/x6941e/x6941e04.htm (accessed July 30, 2019).

5. World Organisation for Animal Health (OIE). Annual report on antimicrobial agents intended for use in animals. Third Report. 2018. http://www.oie.int/fileadmin/Home/eng/Our_scientific_expertise/docs/pdf/AMR/A_Third_Annual_Report_AMR.pdf (accessed July 30, 2019).

6. AACTING: Herd level antimicrobial consumption in animals. http://www.aacting.org (accessed July 30, 2019).

7. OECD/FAO Agricultural Outlook, 2018 - 2027. http://stats.oecd.org (accessed July 30, 2019).
